# Supplementary material for: Emergent neutrality in consumer-resource dynamics
Source: PLoS Comput Biol. 2020 Jul 30;16(7):e1008102. doi: 10.1371/journal.pcbi.1008102 (PMC7446820; doi:10.1371/journal.pcbi.1008102)
Supplement: S1 Appendix — (PDF) [file pcbi.1008102.s001.pdf]

S1 Appendix: Emergent neutrality in consumer-resource dynamics

Rafael D'Andrea [1,*,¶], Theo Gibbs [1,¶], James P. O'Dwyer [1]

Department of Plant Biology, University of Illinois, Urbana IL USA

* rdandrea@illinois.edu

¶ These authors contributed equally to this work.

1 Moment Closure and Mean Field Approximation

In equation (1) of the main text, we propose a set of transition rates defining changes in consumer abundances $\{N_i\}$ and resource concentrations $\{R_k\}$. These define a model with both synchronous and potentially asyncronous depletion of resource k and increase in abundance of consumer i, according to subsitutable preferences C_{ki}. In general, identifying solutions of the master equation defined by these transition rates (Eq. S1 below) is intractable, and so to make progress in understanding the qualitative (and approximately quantitative) features of these solutions, we proposed a comparison of 'deterministic' and drift timescales. In essence, we interpret deterministic here to mean timescales associated with changes in the expectation values of abundances and concentrations. When the changes of the expectation values of abundances are slower (in a sense defined in the main text) than the changes associated with drift, we expect a qualitative transition in both abundance distributions and dynamics of the consumer species.

The master equation is:

$$
\begin{aligned}
\frac{d}{dt}P(\{N_i\},\{R_k\},t) = & \\
& + (\text{rate at which system enters state}\{\{N_i\},\{R_k\}\}) \\
& - (\text{rate at which system leaves state}\{\{N_i\},\{R_k\}\}) \\
= & \\
& + \sum_k P(R_k-1)\; T(R_k-1 \to R_k) \\
& + \sum_k P(N_i+1)\; T(N_i+1 \to N_i) \\
& + \sum_k P(R_k+1)\; T(R_k+1 \to R_k,\; N_i \to N_i) \\
& + \sum_{k,i} P(R_k+1, N_i-1)\; T(R_k+1 \to R_k,\; N_i-1 \to N_i) \\
& - \sum_k P(.)\; T(R_k \to R_k+1) \\
& - \sum_k P(.)\; T(N_i \to N_i-1) \\
& - \sum_k P(.)\; T(R_k \to R_k-1,\; N_i \to N_i) \\
& - \sum_{k,i} P(.)\; T(R_k \to R_k-1,\; N_i \to N_i+1) \\
= & \\
& + \sum_k P(R_k-1)\; \rho_k \\
& + \sum_k P(N_i+1)\; \eta_i(N_i+1) \\
& + \sum_{k,i} P(R_k+1)\; (1-\epsilon)(R_k-1)C_{ki}N_i \\
& + \sum_{k,i} P(R_k+1, N_i-1)\; \epsilon(R_k+1)C_{ki}(N_i-1) \\
& - \sum_k P(.)\; \rho_k \\
& - \sum_k P(.)\; \eta_i N_i \\
& - \sum_{k,i} P(.)\; (1-\epsilon)R_k C_{ki} N_i \\
& - \sum_{k,i} P(.)\; \epsilon R_k C_{ki} N_i
\end{aligned}
\tag{S1}
$$

where for simplicity of notation we only write down explicitly the elements of the system not in state $P(\{N_i\},\{R_k\},t)$ at time t, with $P(.)$ representing the probability that the system is at that state.

To compute the dynamics of expectation values, we need to consider the dynamics of moments of the probability distribution $P(\{N_i\},\{R_k\},t)$ in general, and then make an approximation to discard the effects of non-trivial higher order moments. For the expectation value of resource R_k, we multiply both sides of the master equation defined by Equation (1) of the main text (S1 above) by R_k and then take the expectation value (over $P(\{N_i\},\{R_k\},t)$), leading to:

$$\frac{d\langle R_k\rangle}{dt} = \rho_k + \sum_j C_{kj}\langle R_k N_j\rangle. \tag{S2}$$

Similarly for each consumer abundance N_i, we have that:

$$\frac{d\langle N_i\rangle}{dt} = \epsilon \sum_j C_{ji}\langle N_i R_j\rangle - \eta_i\langle N_i\rangle \tag{S3}$$

To make our mean field approximation, we then assume that $\langle R_k N_j\rangle$ is closely approximated by $\langle R_k\rangle\langle N_j\rangle$, leading to the deterministic dynamics in Equation (2) of the main text.

It is important to note that such approximations can be valid under a range of circumstances, including appropriately-specified large-number and volume limits (e.g. [3]), but will fail in other situations. We do not here seek to delineate precisely the circumstances where our mean field approximation will apply—we simply note that whenever it does, computing timescales associated with expected values of abundances and concentrations becomes much more tractable. Likely the most relevant circumstance where this will be a good approximation in ecology will be where abundances of species are typically large. More generally, one could incorporate higher order moments into a generalized version of Equation (2) of the main text, and solve, but we leave that level of generality for future work.

2 Characterizing the Spectrum of the Deterministic Model

If a system of ordinary differential equations admits a fixed point, then the eigenvalues of its Jacobian evaluated at the fixed point tell us about the behavior of the system after a perturbation. The Jacobian in the system of differential equations in the main text evaluated at the abundances $r\vec{1}$ and $n\vec{1}$ is

$$L = \begin{bmatrix} -n[C\ \vec{1}]_{diag} & -rC \\ \epsilon n C^T & 0 \end{bmatrix} \tag{S4}$$

where $\vec{1}$ denotes a vector of 1's and $[C\ \vec{1}]_{diag}$ denotes a diagonal matrix with entries given by the vector $C\ \vec{1}$. If the real parts of all the eigenvalues of the Jacobian L are negative, then the system will return to equilibrium after a sufficiently small perturbation, and is therefore called locally stable. Previous work has shown that our model is inherently stable [1]. Here, we aim to analytically characterize the eigenvalue distribution of L for both of our parametrizations of C, so we can estimate the rate at which the abundances return to equilibrium.

In Fig A, we plot the spectrum of L in the complex plane and see some characteristic features. There are two complex conjugate eigenvalues, one bulk of eigenvalues near zero and another bulk of eigenvalues centered at $-n\kappa$, where κ is the average row sum of C. In the following sections, we derive approximations to the densities of these two real eigenvalue bulks, and predict the values of the complex conjugate eigenvalues.

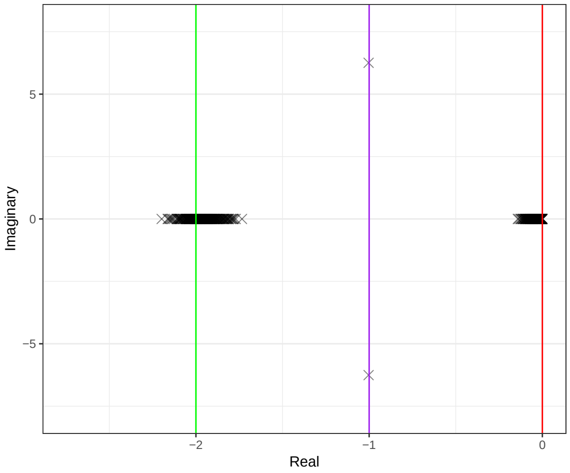

Figure A: The spectrum of L in the complex plane when C is sampled from uniform distribution on $[0, 0.2]$, $K = S = 200$, $n = 1$, $r = 20$ and $\epsilon = 0.5$. The red line is $\Im(z) = 0$, the purple line is $\Im(z) = -\frac{1}{2}\kappa n$ and the green line is $\Im(z) = -\kappa n$.

In Fig B, we plot the magnitude of the components of the eigenvectors corresponding to the two bulks of eigenvalues, and observe that in both the specialist and generalist cases, the eigenvalues near zero largely correspond to consumer directions, while those centered at $-n\kappa$ correspond to resource directions. After any kind of perturbation, we therefore expect resource abundances to quickly decay back to equilibrium, while consumer abundances will decay more slowly, because the eigenvalues which dictate their dynamics are small in magnitude. This timescale separation recapitulates the classic expectation of fast resource dynamics relative to consumer dynamics, but doesn't alone tell us whether the system will be neutral-like, or not.

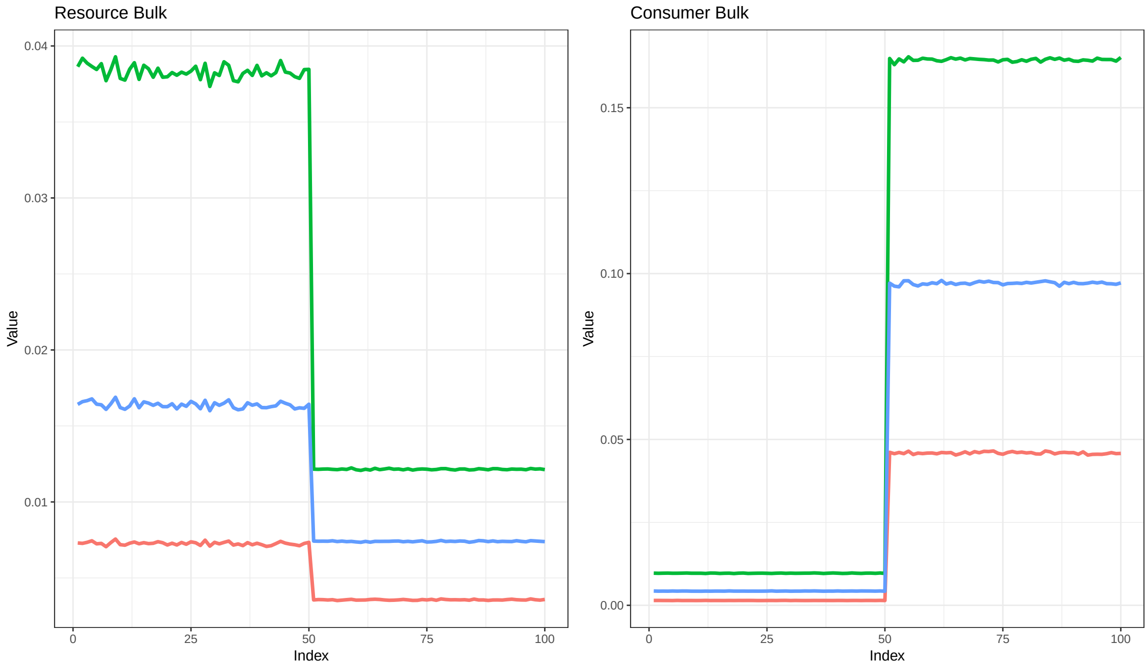

Figure B: The first panel plots the first, second and third quartiles of the absolute values of the components of the eigenvectors associated to the eigenvalues centered at $-n\kappa$, while the second panel plots the same quantities for the eigenvectors associated to the eigenvalues near zero. The first quartile is in red, the median is in blue and the third quartile is in green. For these plots, the entries of the matrix C are sampled from a uniform distribution on $[0.5, 1.5]$, $K = S = 50$, $s = r = 100$ and $\epsilon = 1$. The quartiles are computed using the absolute values of the components of the eigenvectors from 1000 realizations of the matrix C.

3 Master equation and abundance distributions in the neutral and niche limits

Let $Q_k(R_k|\vec{N}, t)$ denote the probability that resource k has abundance R_k at time t in our stochastic model. As mentioned, consumer dynamics are slow, so that $\vec{N}$ is approximately constant in time while resource dynamics play out. The master equation for $Q_k(R_k|\vec{N}, t)$ is then

$$\begin{aligned} \frac{dQ_k(R_k|\vec{N}, t)}{dt} &= \rho_k Q_k(R_k - 1|\vec{N}, t) + \left((R_k + 1) \sum_{j=1}^{S} C_{kj} N_j \right) Q_k(R_k + 1|\vec{N}, t) \\ &- \left(\rho_k + R_k \sum_{j=1}^{S} C_{kj} N_j \right) Q_k(R_k|\vec{N}, t) \; . \end{aligned} \tag{S5}$$

Because of our timescale separation, the sums in Eq S5 are constant, so the stationary distribution is simply a Poisson distribution with rate $\frac{\rho_k}{\sum_j C_{kj} N_j}$. In our simulations, we find that the distribution of a single resource throughout time, and also the distribution of resource abundances across the community, quickly converge to Poisson distributions, in agreement with Eq S5 (see Figs C, D).

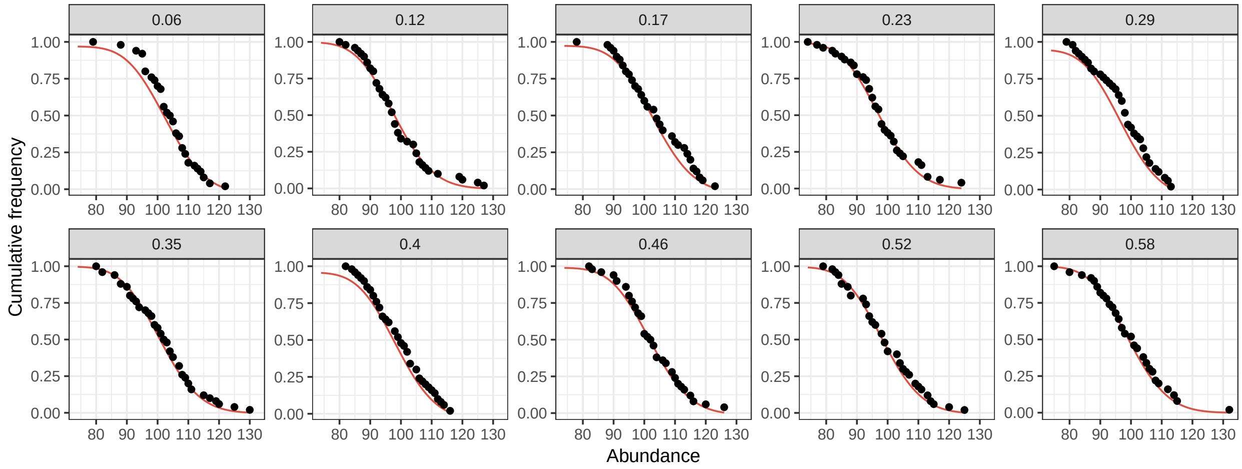

Figure C: Abundance distribution of resources in the generalists scenario. Plots are faceted by the coefficient of variation of the consumption matrix. Red curves show the fitted Poisson distribution. The p-value of the Cramér-von Mises goodness-of-fit test is non-significant for all fits, reflecting successful Poisson fits to resource abundances across the board. Parameters: $n = 500$, $r = 100$, $S = K = 50$.

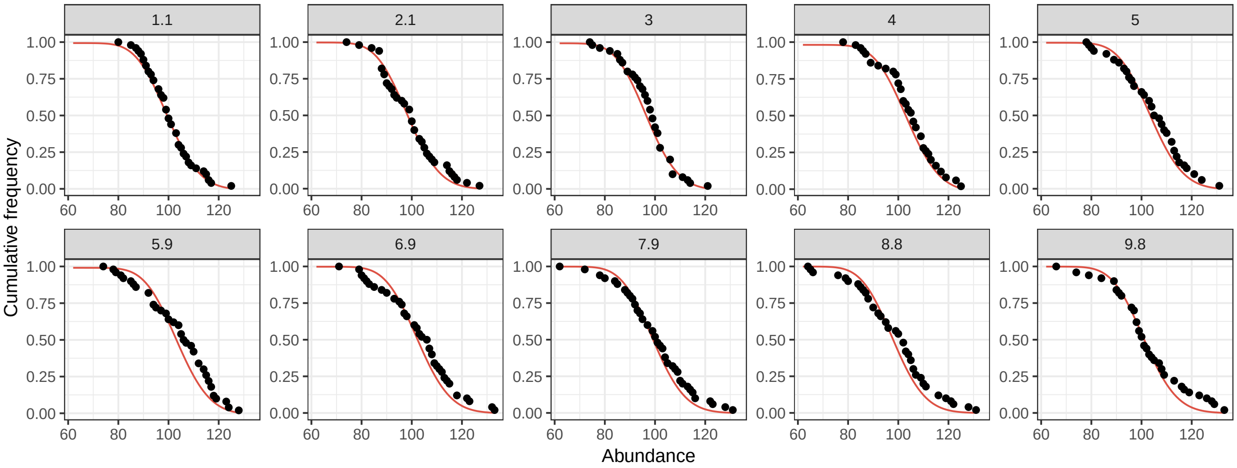

Figure D: Abundance distribution of resources in the specialists scenario. Plots are faceted by the ratio of on-diagonal (preferred resource) to off-diagonal (all other resources) entries in the consumption matrix. Red curves show the fitted Poisson distribution. The p-value of the Cramér-von Mises goodness-of-fit test is non-significant for all fits, reflecting successful Poisson fits to resource abundances across the board. Parameters: $n = r = 100,\ S = K = 50$.

Next, we define $P_i(N_i|\vec{N}, t)$, the distribution of consumer abundance values N_i over an ensemble of trajectories in our stochastic model. Since the resource abundances are tightly concentrated at their mean values, we assume that they are equal to their means, and we eliminate them from the master equation for consumer dynamics, leading to:

$$\begin{aligned}\frac{dP_i(N_i|\vec{N},t)}{dt} &= \epsilon(N_i-1)\left(\sum_{j=1}^{K}\frac{C_{ij}^T\rho_j}{C_{jj}(N_j-1)+\sum_{l\neq j}C_{jl}N_l}\right)P_i(N_i-1|\vec{N},t) \\ &\quad + \eta_i(N_i+1)P_i(N_i+1|\vec{N},t) - N_i\left(\eta_i+\epsilon\sum_{j=1}^{K}\frac{C_{ij}^T\rho_j}{\sum_l C_{jl}N_l}\right)P_i(N_i|\vec{N},t)\ .\end{aligned} \tag{S6}$$

If the consumption coefficients are identical $C_{ij} = \mu$, then the sum $\sum_l C_{jl}N_l = \mu\sum_l N_l = \mu J$ is approximately proportional to the average of the stationary distribution for consumers, where J is the total community size. If J is exactly constant, then Eq S6 becomes the master equation for a neutral birth-death process, with a log-series stationary distribution. More concretely, if $C_{ij} = \mu$, then $\rho_i = \rho$ and $\eta_i = \eta$ for all i and assuming detailed balance gives us the formula

$$P_i(N_i+1) = \frac{\epsilon}{\eta_i}\frac{N_i}{N_i+1}\sum_{j=1}^{K}\frac{C_{ij}^T\rho_j}{\sum_l C_{jl}N_l}P_i(N_i) = \frac{\epsilon S\rho}{J\eta}\frac{N_i}{N_i+1}P_i(N_i) \tag{S7}$$

for all i. Recurring on Eq S7 gives a log-series stationary distribution with parameter $p = \frac{\epsilon S\rho}{J\eta}$ from

the main text. So, when the consumption matrix has all identical coefficients, we expect a log-series stationary distribution as long as the fluctuations of community size J are not prohibitively large at equilibrium. In simulations of the specialist scenario with C_d/C_o varying between 1.1 and 18, the coefficient of variation of the total community size was low throughout our parameter values, never surpassing 0.03, and often as low as 0.01. Therefore, total abundance of consumers was largely invariant, justifying our assumption that J is approximately constant. In this way, we see how neutral drift can plausibly emerge from a consumer-resource model when the differences in consumption preferences among consumers is small. As the coefficients C_{ij} become more heterogeneous, consumer abundances are more strongly affected by specific combinations of consumers, and we expect drift to fail to predict the resulting abundance patterns, because it does not incorporate these relationships. For example, if consumers are completely specialized and C is a diagonal matrix, then $\sum_l C_{jl}N_l = C_{jj}N_j$ is not a good estimate of the average value of the stationary distribution, since it is strongly affected by stochastic fluctuations in N_i at equilibrium. In fact, the stationary distribution for Eq S6 is a Poisson distribution for each consumer in the limit consumers are pure specialists, reflecting the Poisson distribution of their specialized resources.

4 Simplifying the Jacobian

We make the approximation that the upper left hand block of our Jacobian is a diagonal matrix ($-n[C\ \vec{1}]_{diag} \approx -n\kappa I$). When C is specialized, each row sum is precisely $(S-1)C_o + C_d$, so our approximation is exact. When C is random, $\kappa = S\mu$ from the central limit theorem, but each row sum is not exactly constant. Let λ be an eigenvalue of the Jacobian L. If $-(n\kappa + \lambda)I$ is invertible, then we can use block diagonal rules to find the eigenvalues of the Jacobian in Eq S4. On the other hand, if $\lambda = -n\kappa$, then $-(n\kappa+\lambda)I$ is not invertible and we want to find when $\lambda = -n\kappa$ is an eigenvalue of L. Let $\vec{v} \in \mathbb{C}^K$ and $\vec{w} \in \mathbb{C}^S$ be arbitrary vectors. We want to know if $L - \lambda I$ is singular, so suppose

$$\begin{bmatrix} 0 & -rC \\ \epsilon n C^T & -\lambda I \end{bmatrix} \begin{bmatrix} \vec{v} \\ \vec{w} \end{bmatrix} = \vec{0}\,. \tag{S8}$$

Since C is a $K \times S$ matrix and $K \geq S$, C is injective in our parametrizations and hence Eq S8 implies $\vec{w} = \vec{0}$. (C is injective if the equation $C\vec{w} = \vec{0}$ implies that $\vec{w} = \vec{0}$). However, C^T is not injective when $K > S$, so $\lambda = -n\kappa$ is an eigenvalue of the Jacobian L when $K > S$ with multiplicity $K - S$. Now that we understand the case when $\lambda = -n\kappa$, we may safely assume that $\lambda \neq -n\kappa$ and use block determinant rules to predict the remaining eigenvalues. We find that

$$\det[L - \lambda I] = \det[-\kappa n I - \lambda I]\,\det[\epsilon n r C^T((-\kappa n - \lambda)I)^{-1}C - \lambda I]\,. \tag{S9}$$

If we compute the inverse and re-arrange some terms above, we get that the eigenvalues of L satisfy

$$0 = \det[\epsilon n r C^T C - (-\kappa n - \lambda)\lambda I] \ . \tag{S10}$$

Now, let ω be an eigenvalue of $C^T C$. Then,

$$\lambda = -\frac{1}{2}\left(n\kappa \pm \sqrt{(n\kappa)^2 - 4\epsilon n r \omega}\right) \tag{S11}$$

are eigenvalues of L. So, to obtain the spectrum of L, we just need to determine spectrum of $C^T C$. Note that if $S > K$, then $C^T C$ has eigenvalues that are precisely zero and neutrality is assured.

5 Specialist Eigenvalues

In the specialist parametrization, the eigenvalues of $C^T C = C^2$ are simply the eigenvalues of C squared. So, one eigenvalue is the square of the row sum $\kappa^2 = ((S-1)C_o + C_d)^2$ and the remaining eigenvalues are all equal to $(C_d - C_o)^2$. Therefore, we can use Eq S11 to predict the spectrum of L. The resource and consumer eigenvalues are given by

$$\lambda = -\frac{1}{2}\left(n\kappa \pm \sqrt{(n\kappa)^2 - 4\epsilon n r (C_d - C_o)^2}\right) \tag{S12}$$

when the plus-minus is a plus and minus respectively. We use the formula for the consumer eigenvalues to derive the scaling relationship in the main text for the specialist parametrization. The outlying pair of eigenvalues is obtained by applying the transformation in Eq S11 to κ^2, and these eigenvalues will be complex when $4\epsilon r > n$.

For the generalist parametrization, the spectrum of $C^T C$ is more complex. In the following sections, we use random matrix theory to understand the spectrum of L in the generalist case, and then calculate the eigenvalue relevant for predicting the drift threshold.

6 Generalist Outlying Eigenvalues

The Marchenko-Pastur law describes the spectrum of matrices whose entries are independent and identically distributed random variables with mean 0 and variance σ^2. For our application to a consumer-resource model, we need to have $\mu > 0$ so that the entries of C are rates of consumption. Sampling the entries of C from a distribution with non-zero mean changes the

spectrum of $C^T C$ by changing only one eigenvalue. Let's write $C = X + \mu 1_{K\times S}$, where $1_{K\times S}$ is a $K \times S$ matrix of 1s and X is a random matrix with mean 0 and variance σ^2.

$$C^T C = (X^T + \mu 1_{S\times K})(X + \mu 1_{K\times S}) = X^T X + 2\mu X^T 1_{K\times S} + K\mu^2 1_{K\times S} \,. \tag{S13}$$

We expect the row sums of this matrix to be about the same, and so one eigenvector of the matrix should be approximately $\vec{1}$ with an eigenvalue given by the nearly constant row sum. Therefore, the single outlying eigenvalue of a matrix following the Marchenko-Pastur law with non-zero mean should be approximated by $\omega_{\text{out}} \approx S(\sigma^2 + K\mu^2)$. Using the transformation in Eq S11, we find that the two outlying complex conjugate eigenvalues of the spectrum of L are the image of the ω_{out}. Fig E plots the observed and predicted imaginary parts of these outlying eigenvalues of L, while varying μ for different resource abundances r. We have chosen parameters for which $\omega_{\text{out}} = S(\sigma^2 + K\mu^2) > \frac{\kappa^2 n}{4\epsilon r}$ so that the real part of the outlying eigenvalues is simply $-\frac{1}{2}\kappa n$ and the imaginary part is given by the square root in Eq S11. Our predictions work well, and we now turn to the bulks of eigenvalues in the spectrum of L.

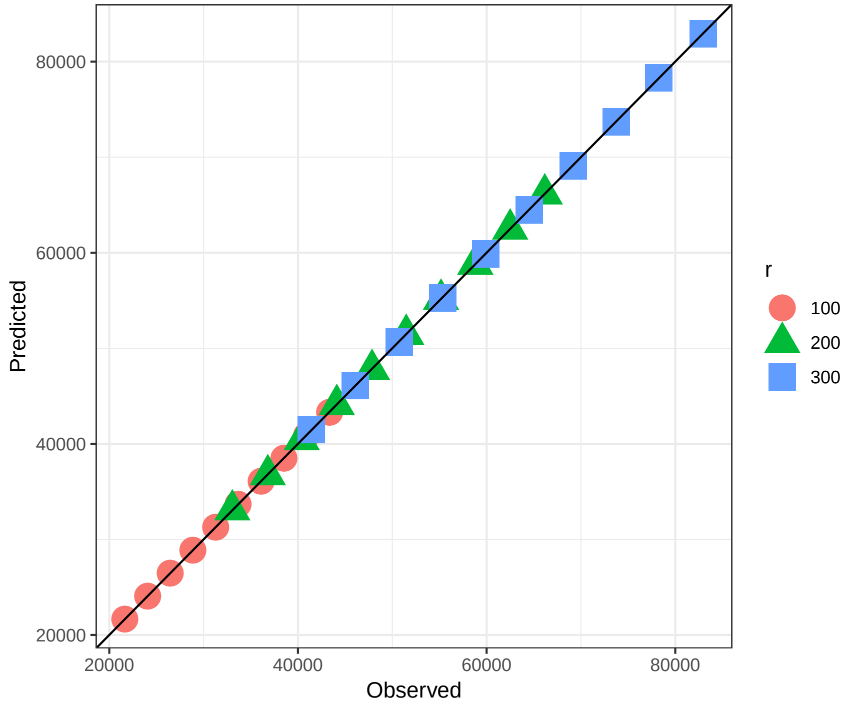

Figure E: The plot shows the observed values of the imaginary part of the outlying eigenvalue of the Jacobian against our analytical prediction while we vary the average consumption strength μ between 0.5 and 1. The C matrix is sampled from a uniform distribution on $[\mu - 0.5, \mu + 0.5]$, $K = S = 500$, $n = 100$, and $\epsilon = 1$. The color and shape of the points denote different r values.

7 Generalist Consumer Eigenvalues

Let X be the matrix from the previous section. Let $\omega_1' \leq \omega_2' \leq ... \leq \omega_K'$ be the K eigenvalues of $\frac{1}{K}X^TX$. Let's define the empirical spectral density of $\frac{1}{K}X^TX$ as

$$\nu_K(z) = \frac{1}{K}\sum_{i=1}^{K}\delta(\omega_i' - z)\ . \tag{S14}$$

In 1967, Marchenko and Pastur proved that if $K, S \to \infty$ such that $S/K \to \gamma \in (0, \infty)$, then $\nu_K \to \nu$ where

$$d\nu(z) = \frac{1}{2\pi\sigma^2}\frac{\sqrt{(\gamma_+ - z)(z-\gamma_-)}}{\gamma z}\mathbf{1}_{[\gamma_-,\gamma_+]}dz \quad \text{with} \quad \gamma_\pm = \sigma^2(1\pm\sqrt{\gamma})^2 \tag{S15}$$

when $\gamma \leq 1$ [2]. $\mathbf{1}_{[\gamma_-,\gamma_+]}$ is the indicator function on $[\gamma_-, \gamma_+]$. When $S > K$ so that $\gamma > 1$, C^TC is not invertible, so there is at least one eigenvalue precisely equal to zero, and we recover the competitive exclusion principle – S species require at least S resources to stably coexist. The density in Eq S15 is called the Marchenko-Pastur law, and is controlled by σ^2, the variance in consumer preferences, and γ, the ratio between the number of consumers and resources. We are interested in the eigenvalues ω of X^TX rather than $\frac{1}{K}X^TX$. Using Eq S15, we see that these eigenvalues are distributed in the interval $[K\gamma_-, K\gamma_+] = [K\sigma^2(1-\sqrt{\gamma})^2, K\sigma^2(1+\sqrt{\gamma})^2]$. So, we understand how the eigenvalues of X^TX are distributed, and now we can use Eq S11 to predict the distribution of the consumer eigenvalues of the full Jacobian. The boundary of the support for the consumer eigenvalues is given by

$$\lambda_\pm = -\frac{\kappa n}{2}\left(1-\sqrt{1-\frac{4\epsilon r}{\kappa^2 n}K\sigma^2(1\pm\sqrt{\gamma})^2}\right) \approx -\epsilon r\frac{\sigma^2}{\gamma\mu}(1\pm\sqrt{\gamma})^2 \tag{S16}$$

where we have expanded the square root to first order to obtain the approximation. λ_+ is the smallest (most negative) eigenvalue that governs local consumer dynamics, so it will correspond to the fastest relaxation time back to equilibrium, and hence it is the eigenvalue we use to derive our analytical prediction for when our stochastic consumer-resource system should appear neutral in the main text. In Fig F, we plot the observed and predicted densities of the consumer eigenvalues of the Jacobian, as well as the predicted value of λ_+, for $\gamma = 1, \frac{1}{2}$. Our predictions work well in each case.

We should note here that we have really computed the eigenvalue distribution of the Jacobian where the upper left hand block is exactly $-n\kappa I$, which could be achieved exactly by, for example, imposing that every row sum of C is the same constant. However, Fig F shows that the prediction for the eigenvalues of this simplified Jacobian still work well for the case of a non-constant upper left diagonal block.

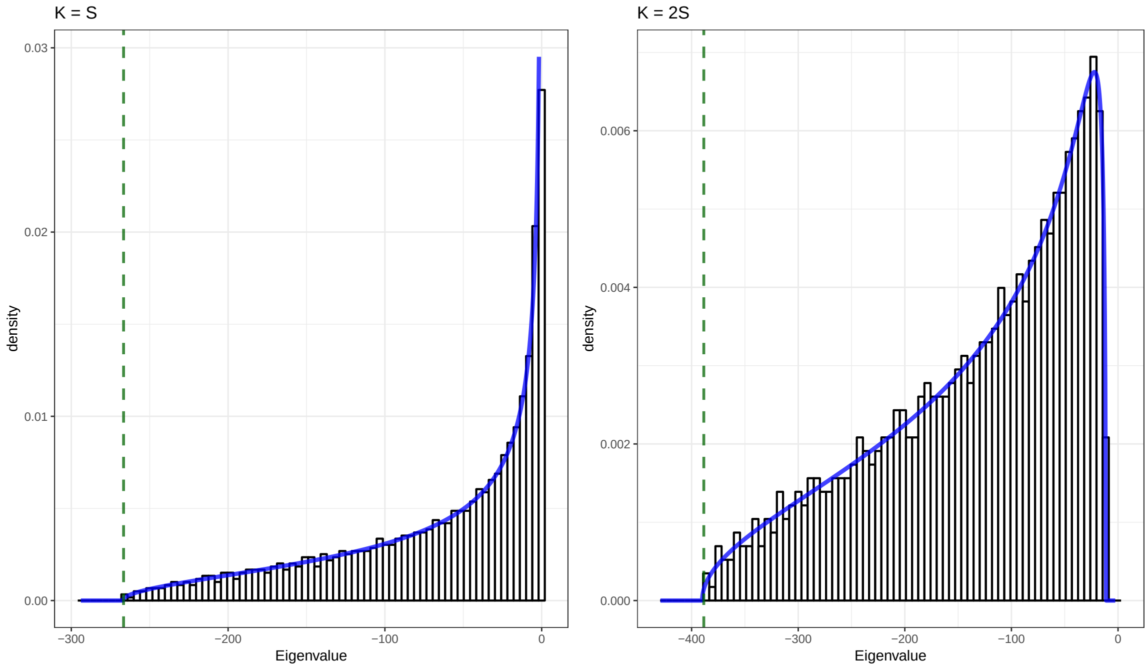

Figure F: The two panels show histograms of the eigenvalues near zero in Fig A for different values of K and S. For both panels, the entries of the C matrix are sampled from a uniform distribution on $[0, 2]$, $s = 100$, $r = 200$ and $\epsilon = 1$. In the first panel, $K = S = 1500$, and in the second panel, $K = 2000$ and $S = 1000$. Our prediction for the density of eigenvalues from Eqs S11, S15 is the curve in blue. The dotted green line is our prediction for λ_+ – the smallest consumer eigenvalue and the eigenvalue we use to predict when the stochastic consumer-resource system should behave neutrally.

8 Generalist Resource Eigenvalues

In contrast to the eigenvalues near zero, the eigenvalues centered at $-n\kappa$ are not well described by the prediction assuming $-n[C\ \vec{1}]_{diag} \approx -n\kappa I$. Here, the heterogeneity in the values of the row sums becomes important for accurately predicting the distribution of resource eigenvalues. Although this is not mathematically justified, Eq S11 still gives us a reasonable approximation to the distribution of resource eigenvalues. We focus on Eq S11 when the $\pm$ is a plus:

$$\lambda = -\frac{1}{2}\left(n\kappa + n\kappa\sqrt{1 - \frac{4\epsilon r}{\kappa^2 n}K\omega'}\right) \approx -n\kappa + \frac{\epsilon r}{\kappa}K\omega'\ . \qquad \text{(S17)}$$

From the central limit theorem, we expect the row sums of C to be normally distributed with mean $S\mu$ and variance $S\sigma^2$. If we take κ to be a random variable with this distribution, we can use Eq S17 to find a new prediction for the resource eigenvalues. In fact, $n\kappa$ is much larger than the ω' term in Eq S17, so it is the distribution of $n\kappa$ that determines the predicted resource eigenvalues.

In Fig G, we plot histograms of the observed resource eigenvalues with the prediction from Eq S17 without the ω term, and find that these predictions still work well. If we normalize the C matrix so that all rows have the same sum and our approximation $-n[C\ \vec{1}]_{diag} \approx -n\kappa I$ is exact, then the resource eigenvalues are described by a Marchenko-Pastur law as predicted by Eq S17, with $K - S$ eigenvalues precisely equal to $-n\kappa$. So, the ω' term in Eq S17 can be important, but, for our parameter choices, the normally distributed row sums of C seem to describe the resource eigenvalues reasonably well.

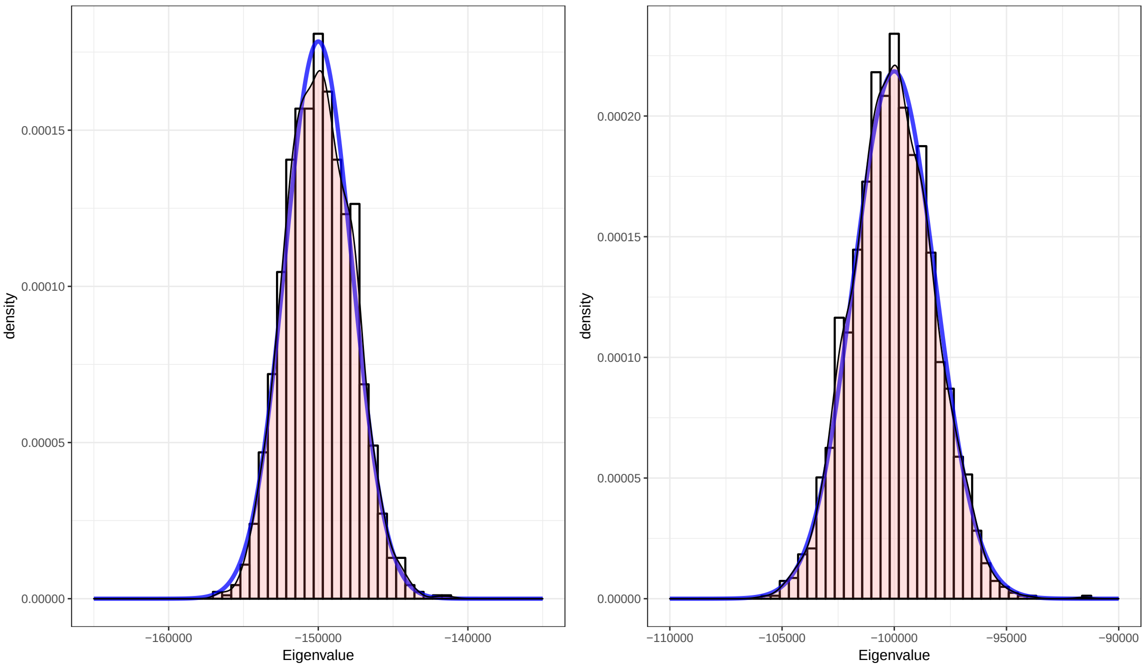

Figure G: The two panels show histograms of the eigenvalues (with the corresponding densities in red) centered at $-n\kappa$ in Figure A for the same parameter values as in Figure F. In the first panel, $K = S = 1500$ and, in the second panel, $K = 2000$ and $S = 1000$. We also plot in blue the prediction from Equation (S17) while neglecting the corrections from the ω' term.

9 Timescale separation between resources and consumers

The timescale separation approximation is important for our master-equation derivations in section 3 above. Thus it may seem that our predictions for drift-like behavior in the consumer community may not apply if this approximation fails. However, we note that the timescale separation arises in precisely the same regimes where we have predicted neutral drift to occur. In fact, these predictions are closely related. From Equation S11, we are predicting that drift will occur when $4\epsilon n r \omega$ is sufficiently small compared to $(n\kappa)^2$ so that the timescale of stabilization is longer than the

timescale of drift fluctuations. Here, ω is the maximum eigenvalue of C^TC and κ is the average row sum of C. In this way, the drift prediction depends on both the eigenvalues of the consumption matrix and other parameters in the problem. Yet when $4\epsilon nr\omega$ is small compared to $(n\kappa)^2$, there is also a natural timescale separation between consumers and resources because some eigenvalues of the Jacobian are small, and we show that the corresponding eigenvectors determine the consumer dynamics. In Figure H below, we plot the average value of the resource and consumer components of the eigenvector corresponding to the small eigenvalues of the Jacobian as a function of the diagonal elements of the consumption matrix C_d in the specialist parametrization for different mean abundances n. As $C_d \to C_o$, we approach true neutrality and the eigenvalues of the Jacobian will truly be zero. In the same limit, the resource components of the eigenvectors approach zero, while the consumer components remain finite. Therefore, the consumer-resource timescale separation is justified precisely when we are predicting drift to be a good description of the dynamics.

The prediction of a timescale separation (and of neutral behavior) still depends on other parameters in the problem, such as the mean consumer abundance, and it is certainly true that the timescale separation is not always a good approximation. For example, if consumers are complete specialists so that each consumes only a single unique resource, then the dynamics of a consumer and its resource are tightly coupled, and it would not be a good approximation to separate their timescales. However, we would not predict the total specialist consumption structure to undergo drift, and therefore it does not matter that the timescale separation is a bad approximation in this regime. In short, the timescale separation in our model emerges as we approach the neutral limit, and it is in this regime where we rely on it for our master-equation analysis.

Put another way, let T_{res} and T_{con} be the characteristic timescales of transient dynamics of resources and consumers, and T_{drift} the characteristic timescale of stochastic drift. Then in the neutral limit, when the niche index is low, we have $T_{\text{res}} \ll T_{\text{drift}} \ll T_{\text{con}}$, and correspondingly we observe neutral behavior among consumers. As we increase the niche index, the resource and consumer timescales change until we reach a regime where $T_{\text{res}} \ll T_{\text{drift}} \sim T_{\text{con}}$, which we identify as the transition point between neutral and niche-like consumer behavior. In both scenarios, the timescale separation between resources and consumers is a good approximation. Far to the right of this regime we have $T_{\text{res}} \sim T_{\text{con}} \ll T_{\text{drift}}$, where the consumer community is decidedly non-neutral and the timescale separation no longer applies.

In sum, the logic of our prediction for the transition point only requires us to calculate stochastic model timescales (i.e. T_{drift}) in the neutral limit, where our simulations show that timescale separation is an excellent description. The rest of the calculation requires only that we calculate the spectrum of the Jacobian, which calculation does not rely on timescale separation, and identify when different timescales cross.

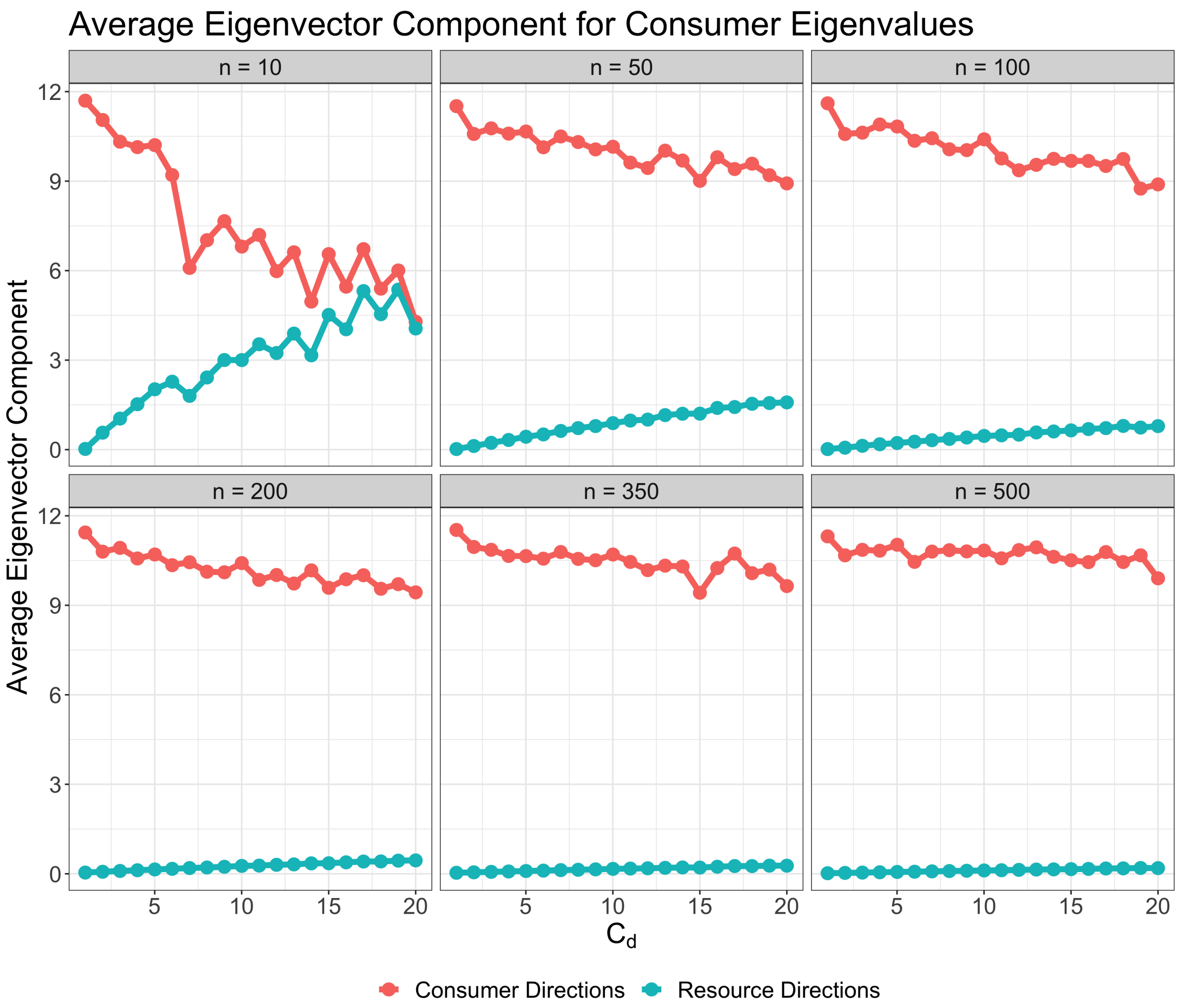

Figure H: Average absolute value of the eigenvector components of the Jacobian corresponding to consumer eigenvalues for different values of the mean abundance, plotted against C_d. Higher C_d indicates higher niche index, i.e. lower neutrality. Consumer eigenvalues are the $S-1$ eigenvalues of the Jacobian that are small as we approach the neutral limit. Blue datapoints show the average across the first S components of the eigenvector (resource directions), while red datapoints show the average across the last S components (consumer directions). We set $r = 100$.

10 Results under alternative methodologies

In the main text, we showed that as the consumption matrix departs from true neutrality, the log-series distribution becomes less likely to fit the species abundance distribution. We defined the point at which this probability falls below 50% as a threshold, which we compared with analytical predictions. While this 50% cutoff is arbitrary, our results are not sensitive to this choice. Specifically, our finding that $CV^{\text{threshold}}$ in the generalist scenario has a power law dependence on the species mean abundance with exponent -0.5 holds whether we define the cutoff at 50% or 5% or 90% (see Fig IA, C). Similarly, our finding that the threshold ratio C_d/C_o in the specialist scenario is linearly related to species richness holds across this wide range of choices for the cutoff (Fig IB, D). This occurs because different cutoff choices lead to thresholds that differ only by a constant multiplicative factor, thus not affecting the functional dependence on n and S.

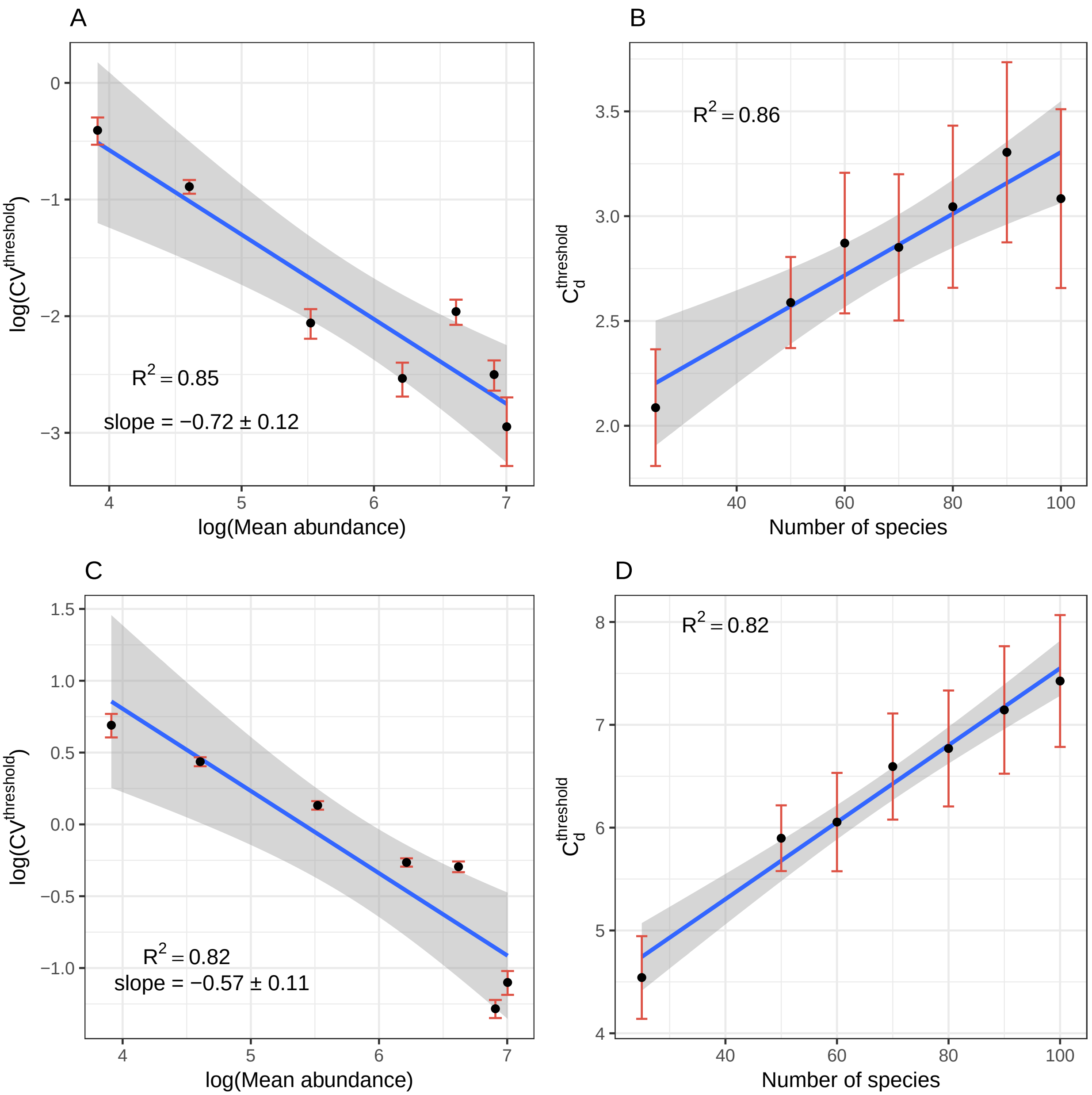

Figure I: The functional dependence of the neutrality threshold on simulation parameters is robust to the choice of the probability cutoff. **A** and **B**: When we set the cutoff to 90% probability of a successful log-series fit, $CV^{\text{threshold}}$ in the generalist scenario (**A**) is still related to mean abundance via a power law with exponent close to -0.5. In the specialist scenario (**B**), the threshold ratio C_d/C_o is still linearly related to the community richness. **C** and **D**: Similar results are obtained when setting the cutoff to 5% probability of successfully fitting the log-series. Compare with Fig. 2C, 2F in the main text. Notice how the slope in B (0.15$\pm$0.05) and D (0.03$\pm$) is different

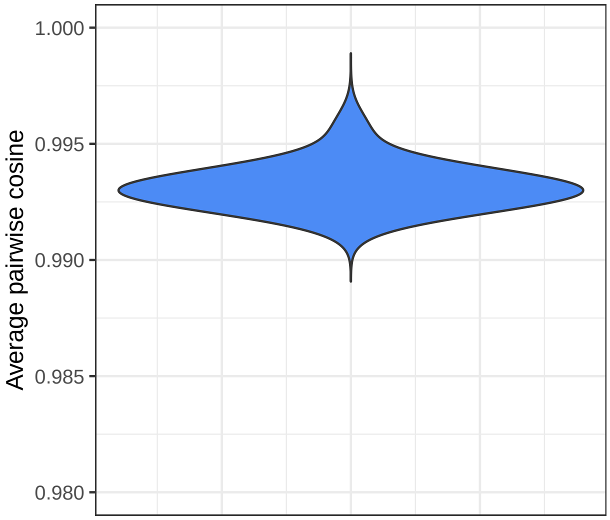

Figure J: Distribution of mean pairwise cosines across an ensemble of neutral communities where every consumption event is recorded over an interval corresponding to the average species lifetime, and then used to estimate the consumption matrix. Namely, the estimated value for matrix entry is $\hat{C}_{ij} = \sum_k \frac{1}{R_{i,k}} \frac{1}{N_{j,k}}$, where $R_{i,k}$ and $N_{j,k}$ are the abundances of resource i and species j at the time of consumption event k, and the sum is over all consumption events involving this pair. From an ensemble of 878 such neutral communities with $S = K = 50, n = r = 100$, the 95% confidence interval for the mean pairwise cosines falls between 0.992 and 0.996. The average species lifetime given these parameters is 250 generations. If consumption events are observed in perpetuity, the estimated consumption matrix converges to the true (neutral) matrix, and the estimated mean pairwise cosine converges to 1.

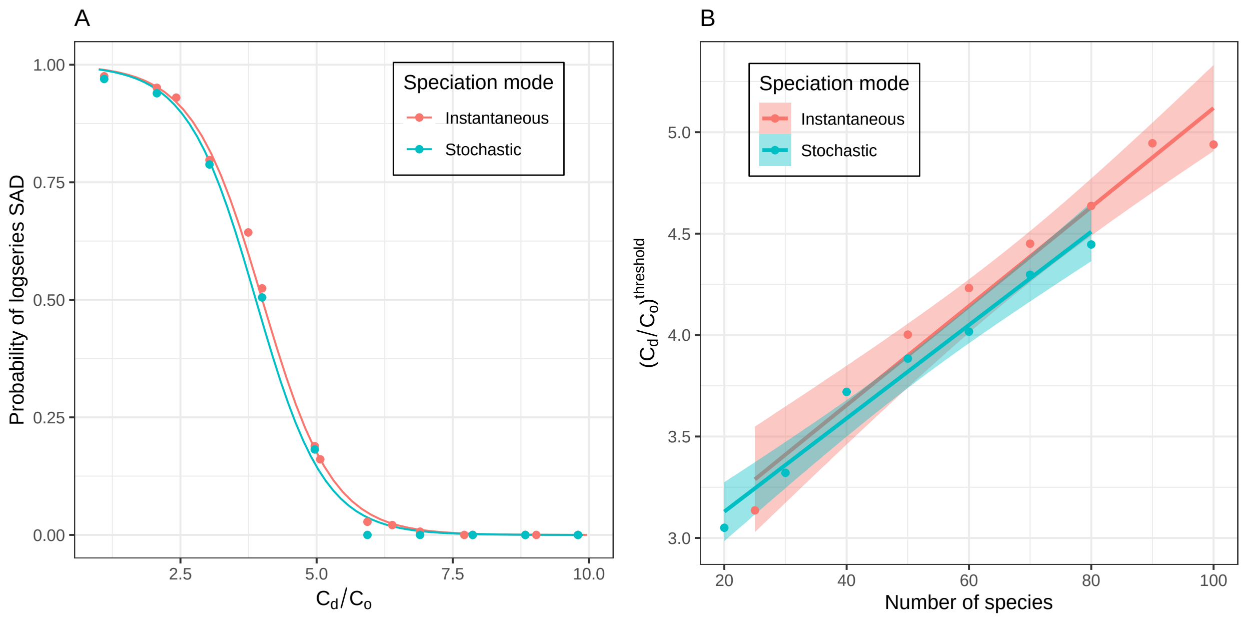

Figure K: Comparison of RSA results between two speciation modes in the specialist scenario. **A**: Probability of a neutral fit plotted against the ratio C_d/C_o of preferred to non-preferred resources, which quantifies the degree of niche differentiation between consumers (Number of species = 50). **B**: Linear behavior of the threshold against the number of species in the community. Results are statistically indistinguishable under either speciation mode (slopes: red: 0.02$\pm$0.002, blue: 0.02$\pm$0.002). Error bars are omitted for visual clarity.

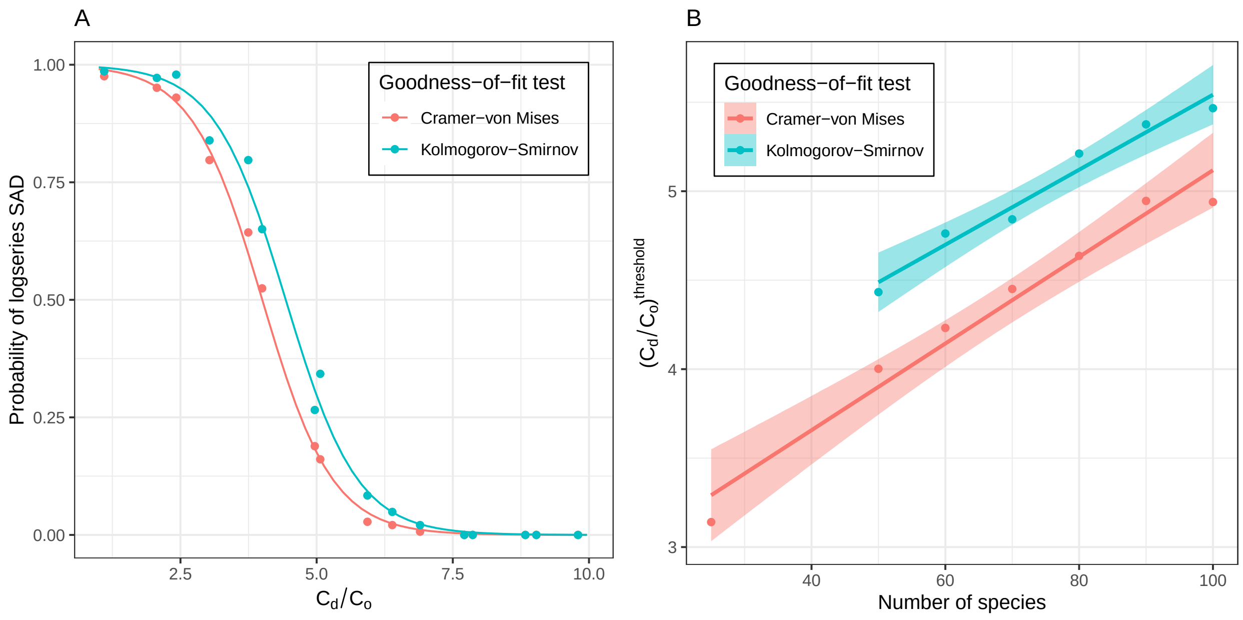

Figure L: Comparison of RSA results between two different goodness-of-fit tests in the specialist scenario. **A**: Probability of a neutral fit plotted against the ratio C_d/C_o of preferred to non-preferred resources, which quantifies the degree of niche differentiation between consumers. Both tests show a quick transition from high to low probability of a neutral RSA, with the threshold value of C_d/C_o, defined as the inflection point in the logistic regression, being slightly different between the two tests. (Number of species = 50). **B**: Both tests reveal linear behavior of the threshold against the number of species in the community. The threshold values are overall higher under the Kolmogorov-Smirnov test, indicating lower power to reject neutrality than the Cramér-von Mises test, however the linear scaling is identical, with indistinguishable slopes: C-vM: 0.02$\pm$0.002, K-S: 0.02$\pm$0.003. (Missing points for the K-S test are due to its being unable to reject neutrality in depauperate communities.)

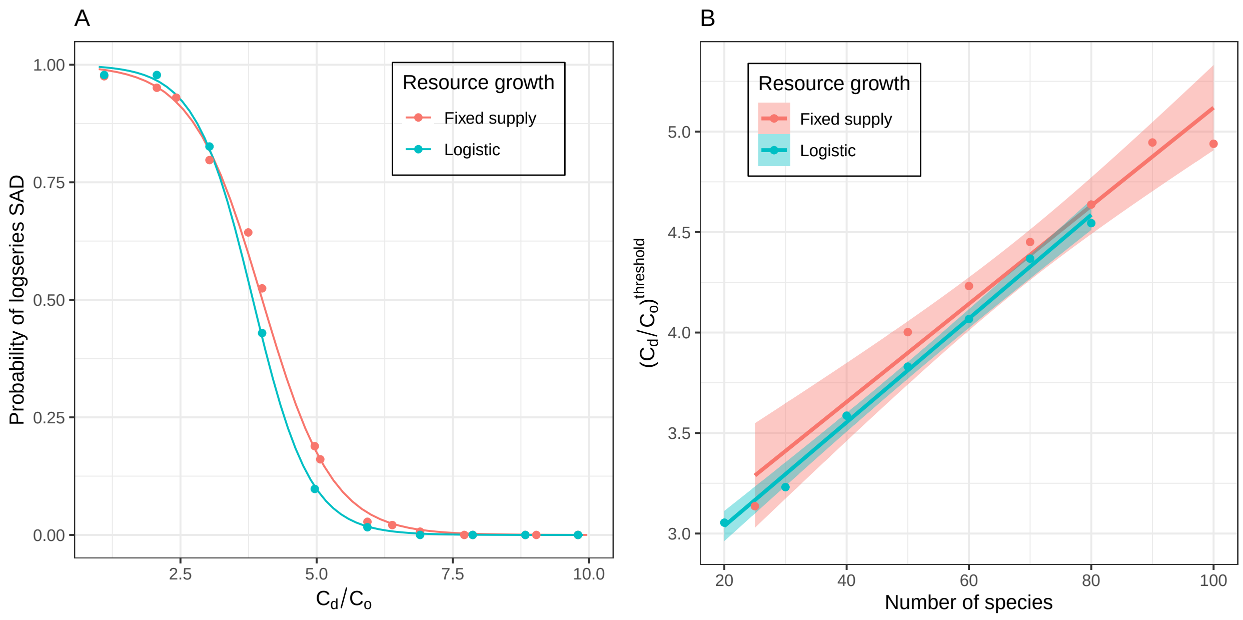

Figure M: Comparison of consumer RSA results in the specialist scenario between two different systems of resource growth: fixed supply and logistic. Under the latter, the probability of a supply event to resource i is proportional to $r_0 R_i(1 - R_i/K)$, where r_0 and K are the intrinsic growth rate and carrying capacity of resources, respectively. **A**: Probability of a neutral fit plotted against the ratio C_d/C_o of preferred to non-preferred resources, which quantifies the degree of niche differentiation between consumers. Both scenarios show a quick transition from high to low probability of a neutral RSA (Number of species = 50). **B**: The logistic scenario also shows linear behavior of the threshold against the number of species in the community (R^2 = 0.97). Slopes are similar between the two scenarios: fixed supply: 0.02±0.002, logistic supply: 0.03±0.0008.

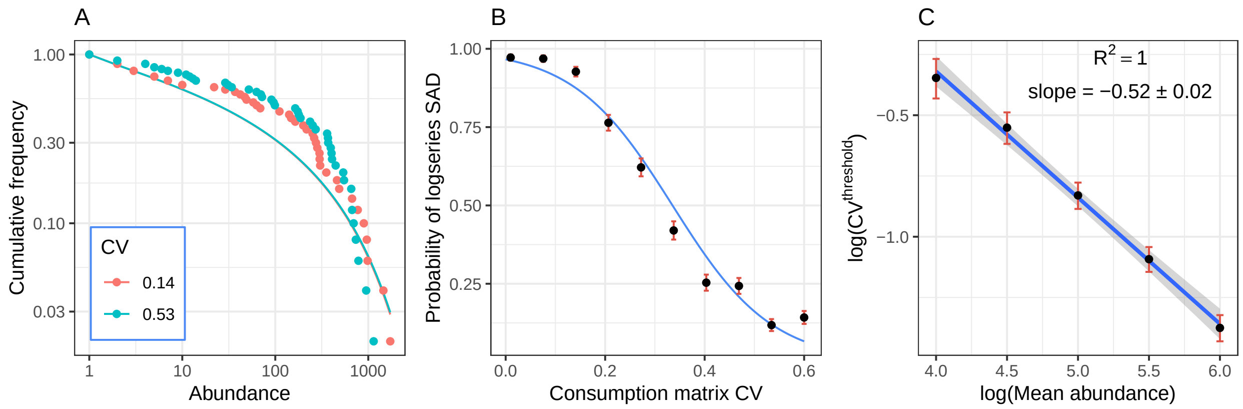

Figure N: SAD results for the generalist scenario where the C matrix is drawn from a normal distribution. Results are analogous to those for C drawn from a uniform distribution presented in Figs 2A, B, C in the main text. **A**: The SAD fits a log-series (solid curve) when the coefficient of variation (CV) in the C matrix is sufficiently low (red points, Cramér-von Mises test p-value = 0.12), but not when the CV is sufficiently high (blue points, Cramér-von Mises test p-value = 0.005). **B**: The probability of a neutral fit is high for low coefficient of variation (CV) in the C matrix, and quickly decreases as the CV increases, with an inflection point at CV = 0.33. Points and error bars show the mean and standard error of the count of successful fits, out of an ensemble of 288 communities. Blue curve shows logistic regression. (Number of species = 50; Mean abundance = 245) **B**: The threshold CV, defined as the inflection point of the logistic regression, has a power-law dependence on the mean species abundance with fitted exponent -0.52±0.02, matching our prediction of -0.5.

11 Behavior of the niche-neutral transition as a function of diversity and community size

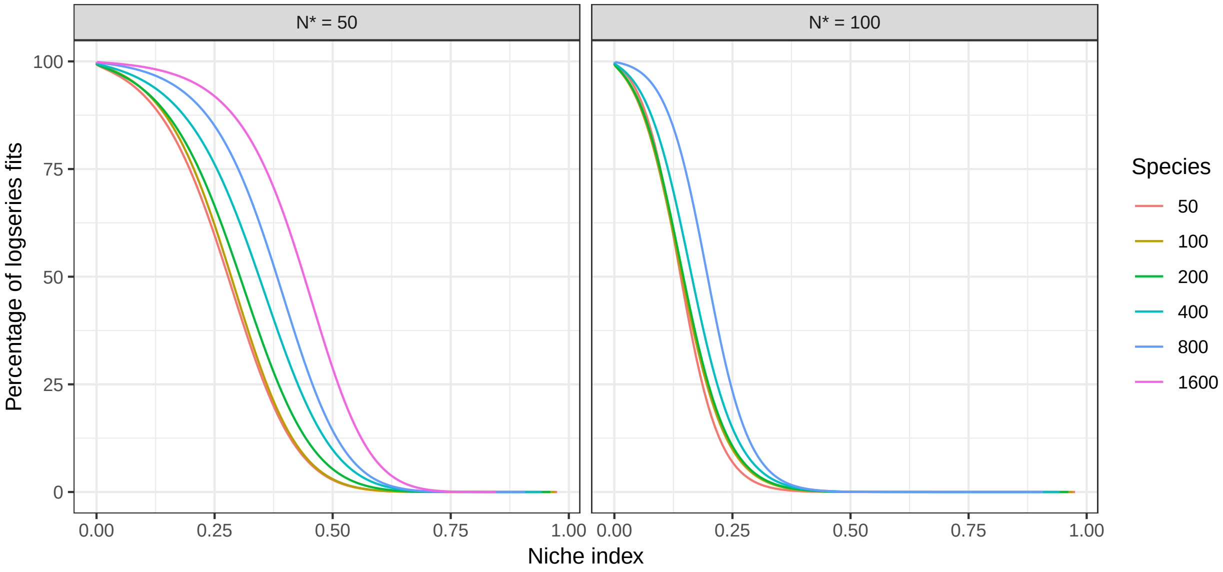

Figure O: Transition between drift-like and niche-like abundance distributions, as represented by the percentage of communities with logseries SAD, plotted as a function of the niche index of the resource-consumer matrix. The niche index is defined as $1 - \mathrm{mean}(\cos(C_{i.}))$, where $C_{i.}$ is the i-th row of the consumer-resource matrix indicating the dietary preferences of species i. While the location of the midpoint in the transition between high and low likelihood of a logseries SAD depends on the number of species in the community (as explored in detail in the main text), the width of the transition is relatively unaffected. However, the curves are steeper/narrower when the community size is larger (compare right panel to left panel). Curves show fitted logistic regressions to sets of 200 communities for each of 25 values of the niche index.

References

[1] Butler, S. and O'Dwyer, J. P. (2018). Stability criteria for complex microbial communities. *Nature Communications*, 9.

[2] Marčenko, V. A. and Pastur, L. A. (1967). Distribution of eigenvalues for some sets of random matrices. *Mathematics of the USSR-Sbornik*, 1(457).

[3] van Kampen, N. (2007). *Stochastic processes in physics and chemistry*. Elsevier, Amsterdam.
